# Combining Model‐Based Clinical Trial Simulation, Pharmacoeconomics, and Value of Information to Optimize Trial Design

**DOI:** 10.1002/psp4.12579

**Published:** 2020-12-31

**Authors:** Daniel Hill‐McManus, Dyfrig A. Hughes

**Affiliations:** ^1^ Centre for Health Economics and Medicines Evaluation Bangor University Bangor UK

## Abstract

The Bayesian decision‐analytic approach to trial design uses prior distributions for treatment effects, updated with likelihoods for proposed trial data. Prior distributions for treatment effects based on previous trial results risks sample selection bias and difficulties when a proposed trial differs in terms of patient characteristics, medication adherence, or treatment doses and regimens. The aim of this study was to demonstrate the utility of using pharmacometric‐based clinical trial simulation (CTS) to generate prior distributions for use in Bayesian decision‐theoretic trial design. The methods consisted of four principal stages: a CTS to predict the distribution of treatment response for a range of trial designs; Bayesian updating for a proposed sample size; a pharmacoeconomic model to represent the perspective of a reimbursement authority in which price is contingent on trial outcome; and a model of the pharmaceutical company return on investment linking drug prices to sales revenue. We used a case study of febuxostat versus allopurinol for the treatment of hyperuricemia in patients with gout. Trial design scenarios studied included alternative treatment doses, inclusion criteria, input uncertainty, and sample size. Optimal trial sample sizes varied depending on the uncertainty of model inputs, trial inclusion criteria, and treatment doses. This interdisciplinary framework for trial design and sample size calculation may have value in supporting decisions during later phases of drug development and in identifying costly sources of uncertainty, and thus inform future research and development strategies.

**Study Highlights**

**WHAT IS THE CURRENT KNOWLEDGE ON THE TOPIC?**

☑ A Bayesian decision‐theoretic approach to sample size calculation provides an alternative to the more traditional methods based on thresholds for type I and type II error probabilities. Prior distributions for treatment effects are required, but these may be biased if based solely on the results of previous trials.

**WHAT QUESTION DID THIS STUDY ADDRESS?**

☑ If estimates of treatment effects from previous studies are not suitable, can pharmacometric model‐based clinical trial simulation be used to generate prior distributions of treatment effects for Bayesian trial design?

**WHAT DOES THIS STUDY ADD TO OUR KNOWLEDGE?**

☑ This study provides a demonstration of the steps involved in simulating trial outcomes, for a range of trial designs, and using these to optimize trial design based on maximizing return on investment.

**HOW MIGHT THIS CHANGE DRUG DISCOVERY, DEVELOPMENT, AND/OR THERAPEUTICS?**

☑ This method may facilitate or enhance Bayesian clinical trials design in situations where changes in treatment dose, regimen, patients, or comparators, means that evidence from earlier studies is not likely to provide a reliable estimate of treatment effect in future studies.

The principal objectives of phase III clinical trials are to confirm efficacy and assess the benefit‐risk ratio in order to gain regulatory approval.^1^, ^2^ The evidence gained in this phase also forms the basis for health technology assessment, and decision‐making regarding reimbursement.^3^ The design of phase III trials, specifically in relation to sample size calculations, has conventionally used power calculations based on thresholds for type I and type II statistical errors and estimates of minimal clinically important differences and variances in treatment effect.^4^, ^5^ There are well known limitations with this approach, in particular that the thresholds for type I or type II error are arbitrary and do not take into account the cost associated with making these errors.^6^ Furthermore, the focus is on passing the regulatory hurdle, even though pricing and reimbursement decisions will also be determined by the evidence that is generated in this phase.

Bayesian methods provide the main alternatives to the more traditional approach to sample size calculation and can be classed as either inference‐based or taking a decision‐theoretic approach.^7^ From a decision‐theoretic, or fully Bayesian,^8^ perspective the value of collecting additional data is that it may reduce the probability of incorrectly making a suboptimal decision with respect to some utility function, thus incurring an opportunity cost. The data derived from larger samples therefore has value that must be weighed against the cost required to obtain the data.^9^ Much work has been done to further develop this methodology so that it can be applied in a wide range of contexts,^10^, ^11^ and extended to various decision perspectives,^12^, ^13^ however, real‐world applications remain limited.^14^

From a pharmaceutical industry perspective, larger studies should yield more precise estimates of treatment effects thereby reducing the uncertainty associated with achieving regulatory approval and reimbursement. Payers in many jurisdictions consider the cost‐effectiveness of new pharmaceuticals during the reimbursement decision‐making process,^15^ with more effective and less costly drugs more likely to be reimbursed. If payer decision‐making makes use of cost‐effectiveness thresholds, for example,^16^ then there is also a relationship between the observed treatment effects in pivotal studies and the maximum price at which the payer would support reimbursement. This relationship is often informed via a cost‐effectiveness analysis based on pharmacoeconomic modeling.^17^

The Bayesian decision‐analytic approach to trial design uses prior distributions for treatment effects, which are then updated based on likelihood models for the data coming from a possible trial. Priors could be based on previous, such as phase II, trial results^13^, ^18^, ^19^ or on elicited expert opinion.^20^ These methods have their limitations, including the potential for bias with expert elicitation^21^ and sample selection bias for previous trials.^22^ Furthermore, there will be added difficulties in making use of past trial data if proposed trial designs are expected to differ in the characteristics of patients, medication adherence,^23^ or are using a different dose or regimen to that previously investigated.

A possible method of incorporating evidence from, potentially multiple, earlier phase studies that has not previously been applied in a Bayesian decision‐theoretic context, is to make use of pharmacometric‐based clinical trial simulation (CTS).^24^ Pharmacometric modeling and simulation is used routinely during drug development, including to study issues relating to the design of clinical trials.^25^, ^26^, ^27^, ^28^, ^29^ The advantage of pharmacometric‐based CTS is that it can account for subject‐specific covariates, imperfect medication adherence, alternative doses and regimens, and can be used to simulate the comparator arm(s) of the proposed trial. The uncertainty in parameter estimates that is quantified during model development can be used in Monte Carlo simulation to generate a distribution of trial outcomes representing prior belief, but based on specific doses, regimens, comparators, and patient population, while also adjusted for protocol deviations, such as imperfect medication adherence.^24^

The aim of this study was to demonstrate the utility of using CTS to generate prior distributions for use in Bayesian decision‐theoretic sample size calculation. Sample sizes are optimized with respect to the pharmaceutical company return on investment (ROI), and drug price is linked to the outcome of a clinical trial via a pharmacoeconomic model representing the perspective of a reimbursement authority. The process is illustrated using a case study of an already marketed drug used to treat hyperuricemia in patients with gout. We show that the approach can be applied to study a variety of design issues, apart from sample size, including trial inclusion/exclusion criteria, duration, drug adherence, or discontinuation.

## METHODS

This study takes the perspective of a pharmaceutical company planning for phase III testing of a drug ahead of submission for marketing authorization. As a case study, using a drug with known pharmacokinetics (PK) and pharmacodynamics (PD), and with completed phase III trials and pharmacoeconomic evaluations, we have used the urate‐lowering therapy febuxostat, which is already marketed for the treatment of gout. The aim of urate‐lowering therapy, both in clinical trials and in routine practice, is to reduce serum uric acid (sUA) concentration to below 6 mg/dL, which should lead to the dissolution of crystals and reduction or elimination of gout symptoms. There were four phase III trials of febuxostat (once‐daily doses ranging between 40 and 240 mg) versus the standard of care, allopurinol (once‐daily doses ranging between 100 and 300 mg). However, for simplicity, we have only considered the design of a single two‐arm trial of febuxostat 80 mg versus allopurinol 300 mg.

The following will describe a simulation framework combining a pharmacometric CTS, a pharmacoeconomic model, and a model of the company’s ROI applied to compare trial designs and perform sample size calculations. It is assumed that the pharmaceutical company has developed the necessary pharmacometric models from early phase studies, which are capable of simulating the relevant phase III trial end points. These models characterize the dose‐response relationships, covariate effects, and quantify sources of uncertainty, including for model parameters. The CTS was used to generate distributions of treatment effects, for a specific patient population and under specific dose‐taking conditions, which were then used as prior distributions in a Bayesian decision‐theoretic sample size calculation.

### Clinical trial simulation model

The CTS consisted of linked PK and PD models for both allopurinol and febuxostat, as well as a trial execution model. The PK for allopurinol and febuxostat were described using one‐compartment and two‐compartment models, respectively. The PD model consisted of a multicompartment, semi‐mechanistic model of uric acid production and renal excretion. The drug PD models used inhibitory indirect response equations, with febuxostat having an additional stimulatory impact on the renal excretion of the uric acid precursor xanthine. Given the individual dosing histories of trial subjects, the PK/PD model was used to simulate sUA trough concentrations on each day at the time of dose administration. Details of the PK/PD model development have been published previously^30^, ^31^ and more details are provided in the **Supplementary Material**.

The trial execution model includes the trial duration, inclusion/exclusion criteria, recruitment, and drug adherence. Both arms were populated by random sampling from attribute distributions representing the gout population (from previous trials) and application of inclusion/exclusion criteria (**Supplementary Material**). These attributes included subjects’ baseline sUA concentration, body weight, and age, which are covariates in the PK/PD model. Drug adherence comprises the initiation of treatment, the degree to which a patient’s dose taking matches the prescribed regimen while nominally adhering (implementation), and treatment discontinuation.^32^ It was assumed that all patients initiate treatment and patients who discontinue revert to their baseline sUA concentration. Implementation was modeled according to a subject‐specific probability of taking each dose, independent of whether any previous doses were taken. The population dose implementation probability was assumed to have a mean of 0.9. Discontinuation was simulated using a daily hazard, modeled as a Weibull hazard function such that the risk of discontinuation falls over time.

In order to propagate uncertainty in input parameters to predicted uncertainty in the trial outcome, each CTS was replicated 10,000 times with resampling from input parameter probability distributions. The variance‐covariance matrix for model parameters would typically be estimated during the development of the PK/PD model. For the purpose of demonstrating this methodology we have assigned arbitrary variances to model parameters to examine two different scenarios: (i) the base case in which parameters are assumed to be highly uncertain and (ii) a reduced uncertainty scenario with lower variances for all PK/PD parameters. The reduced uncertainty scenario is used to represent the option of temporarily foregoing a phase III trial in favor of a smaller, shorter study designed to reduce uncertainty surrounding the drug pharmacology. Parameter uncertainty was simulated using a constant coefficient of variation model, for example, ${\text{KA}}_{i}=\text{KA}*e^{\eta_{{\text{KA}}_{\text{i}}}}$ is the absorption in the *i*th CTS simulation and $\eta_{\text{KA}}$ is a random normal variate with mean of zero and assumed variance. Each iteration of the CTS used 1,000 subjects in order to minimize any variation due to sampling the trial cohort, and did not simulate intra‐individual variability. Complete tables of all CTS model parameters and coefficients of variation and interindividual variability models are given in the **Supplementary Material**.

Apart from the consideration of two scenarios for PK/PD parameter uncertainty, we have also examined three possible dose and inclusion criteria options for each trial arm, as summarized in **Table** **1**. Designs $\psi_{2}$ and $\psi_{5}$ using allopurinol 300 mg and febuxostat 80 mg with a minimum baseline sUA concentration of 8 mg/dL, for inclusion in the trial, are most aligned with the previous trials. Designs $\psi_{1}$ and $\psi_{4}$ use a lower the cutoff of 6 mg/dL and designs $\psi_{3}$ and $\psi_{6}$ use a lower cutoff in conjunction with higher doses of both drugs. The result of the CTS is a distribution of the primary trial outcome $\theta_{\mathrm{jk}}$, and the percentage of subjects with a final sUA concentration of < 6 mg/dL, for a treatment *j* and trial design *k*.

**Table 1:** Overview of 12 clinical trial simulations performed using two treatment arms and six trial designs

Arm	Scenario	Design	Dose, mg	Duration, days	sUA threshold, mg/dL
Allopurinol	Base case	ψ_1_	300	182	6
		ψ_2_	300	182	8
		ψ_3_	600	182	6
	Reduced uncertainty	ψ_4_	300	182	6
		ψ_5_	300	182	8
		ψ_6_	600	182	6
Febuxostat	Base case	ψ_1_	80	182	6
		ψ_2_	80	182	8
		ψ_3_	120	182	6
	Reduced uncertainty	ψ_4_	80	182	6
		ψ_5_	80	182	8
		ψ_6_	120	182	6

sUA, serum uric acid.

**Figure 1:**
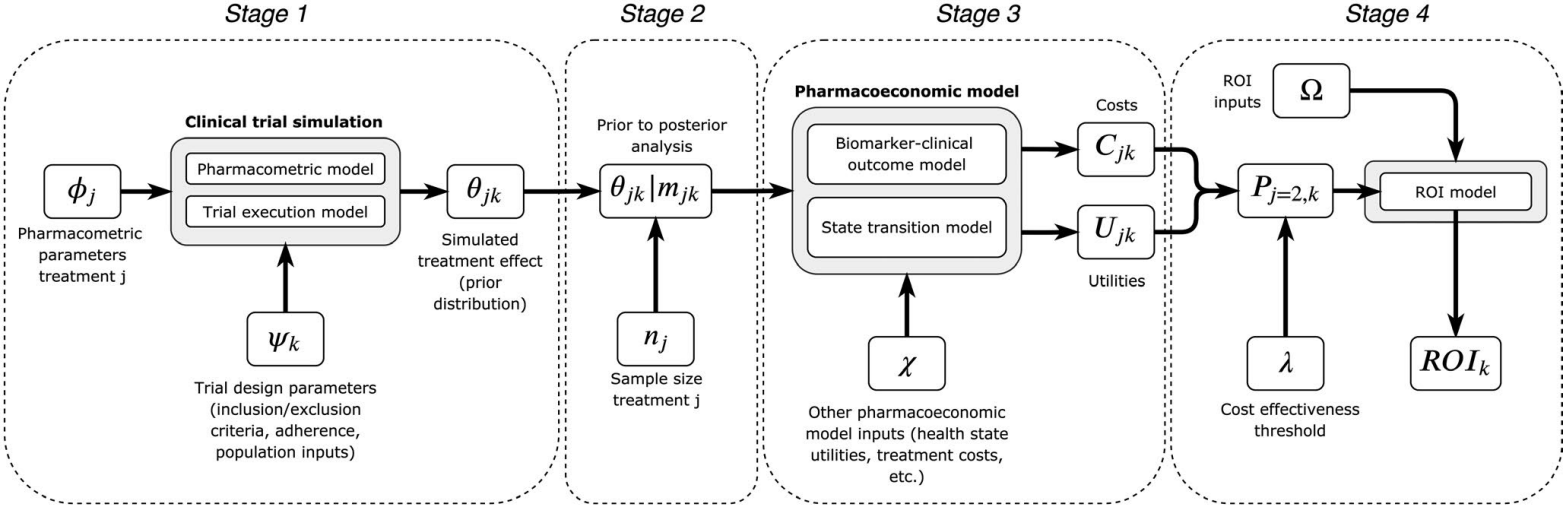
Representation of the simulation framework showing the four stages and key inputs and outputs at each stage. Stage 1: clinical trial simulation; stage 2: Bayesian updating (prior to posterior analysis); stage 3: pharmacoeconomic modeling; and stage 4: implementation of the ROI model. The procedure is replicated a large number of times with resampling from pharmacometric and trial design parameters such that subsequent outputs are in fact distributions. The subscript *j* indexes over the trial arms and the subscript *k* indexes over the trial designs. ROI, return on investment.

The CTS, represented by stage 1 in **Figure** **1**, was performed using R version 3.5.1 and implemented on the Supercomputing Wales cluster to enable parallelization. The 10,000 CTS replicates for a given trial design were split into parallel groupings that were then run in series using 480 CPUs. A single model simulated both a febuxostat and an allopurinol arm, therefore, six models were used that correspond to the trial designs in **Table** **1**.

### Bayesian updating

In the case where the outcome of interest is a proportion, in this case the proportion of subjects achieving a reduction in sUA to below 6 mg/dL, we may model the outcome in one arm of a clinical trial using the binomial distribution m∼Bin(n,θ), where θ is the probability of treatment success (sUA < 6 mg/dL) and *n* is the sample size. The probability of treatment success will be a function of the pharmacology of the drug (e.g., its potency) and of the trial design (e.g., subject attributes) so may further write: $m_{j}∼\text{Bin(}n_{j},\theta_{j}\left(\psi ,\phi_{j}\right))$ where ψ is a specified trial design, ϕ is the drug pharmacology, and the subscript *j* refers to a specific trial arm/treatment, *j *= {1,2}.

We treat the CTS results as the prior distribution $\theta_{j}$, representing our belief regarding the probability of treatment success based on prior knowledge of the drugs’ pharmacology and of dose‐taking behavior. The computation of the posterior distribution $\theta_{\mathrm{jk}}|m_{\mathrm{jk}}$ would be straightforward for a conjugate prior with a Beta distribution. The subscript *k* has been included to index over a set of possible trial designs, $\psi =\{\psi_{1},\psi_{2},\ldots ,\psi_{6}\}$. To facilitate Bayesian updating in this case study, we used the simulated means and variances to calculate the corresponding α and β parameters of Beta distributions.

The posterior distribution of the treatment effect can then be written, for the *j*th trial arm and the *k*th trial design, as:

(1) $$\theta_{\mathrm{jk}}|m_{\mathrm{jk}}∼B\left(\alpha_{\mathrm{jk}}+m_{\mathrm{jk}},\beta_{\mathrm{jk}}+n_{j}‐m_{\mathrm{jk}}\right)$$

For each CTS‐generated Beta prior, posterior densities were computed for a range of different proposed sample sizes in each trial arm $n_{j}=\left\{50,100,150,200,300,400,500,600\right\}$.

### Maximum reimbursement pricing

This framework also considers the perspective of a reimbursement authority and how their decision on reimbursement is a function of both drug price and clinical trial outcome. To facilitate the analysis, a single‐payer healthcare service (the National Health Service in the United Kingdom) was considered, but other payers or multiple payer models could also be developed. Cost‐effectiveness is central to health technology appraisals in the United Kingdom and is assessed via economic evaluation that estimates the long‐term costs and benefits of adopting a new drug.^33^ A cost‐effectiveness threshold of £20,000–£30,000 per quality‐adjusted life year (QALY) is used, and medicines are deemed cost‐effective if estimated to result in a positive incremental net monetary benefit (NMB). Incremental NMB is defined as $\Delta B=\lambda \left(Q_{2}‐Q_{1}\right)‐\left(C_{2}‐C_{1}\right)$, where λ is the payer’s cost‐effectiveness threshold, $Q_{2}‐Q_{1}$ is the incremental QALYs gained, and $C_{2}‐C_{1}$ is the incremental cost. Economic evaluations typically use pharmacoeconomic models to estimate costs and QALYs over an appropriate time horizon and for the relevant patient population. We have applied a pharmacoeconomic model in order to interpret proposed trial evidence from the reimbursement authority perspective and calculate the maximum they should be willing to pay (maximum reimbursement price (MRP)) to provide patient access to a drug providing benefit θ|m.

As illustrated in **Figure** **1**, $Q_{j}$ and $C_{j}$ are functions of the posterior outcomes for each treatment and design $\theta_{\mathrm{jk}}|m_{\mathrm{jk}}$ and the other pharmacoeconomic model inputs (χ). We implemented a previously published pharmacoeconomic model that links sUA concentration subgroupings to acute gout flare frequency to estimate long term QALYs and costs.^31^ The economic model used a Markov state‐transition structure with a 3‐month time cycle, a lifetime (50 year) time horizon, discounting of costs and QALYs at a rate of 3.5% per annum,^33^ and assumed a starting cohort of patients with untreated gout representative of the United Kingdom. The model predicts the impacts of two alternative payer decisions; (i) febuxostat recommended as first‐line therapy, or (ii) febuxostat NOT recommended as first‐line therapy and instead continue to treat all patients with allopurinol. Economic model inputs other than treatment response rates were not varied during simulations, assuming that reimbursement decisions are based on expected values of these inputs.^34^

From the pharmacoeconomic model, therefore, we obtain distributions of $Q_{j}$ and $C_{j}$ that are functions of the posterior outcomes for each treatment and other pharmacoeconomic model inputs (**Figure** **1**):

(2) $$\begin{array}{c} Q_{\mathrm{jk}}∼f\left(\theta_{\mathrm{jk}}|m_{\mathrm{jk}},\chi \right)\\ C_{\mathrm{jk}}∼f\left(\theta_{\mathrm{jk}}|m_{\mathrm{jk}},\chi \right) \end{array}$$

where *j* = 1 for allopurinol and 2 for febuxostat. Then, by separating febuxostat cost into drug and non‐drug components, setting NMB to zero and rearranging for the price of febuxostat ($P_{2}$) we can obtain for the *k*th trial design:

(3) $$P_{2}=\frac{C_{1}\left(\theta_{1}|m_{1},\chi \right)‐{\bar{C}}_{2}\left(\theta_{2}|m_{2},\chi \right)+\lambda \left(Q_{2}\left(\theta_{2}|m_{2},\chi \right)‐Q_{1}\left(\theta_{1}|m_{1},\chi \right)\right)}{t_{2}\left(\psi \right)}$$

In the equation above, the subscripts shown refer to the trial arm *j*. All variables except χ should have a *k* subscript for the trial design, this has been omitted to aid the presentation. The *t*_2_ is the mean number of years for which a patient is expected to persist with febuxostat and is a function of the rate of dropout used in the pharmacometric model, and ${\bar{C}}_{2}$ is the expected cost impact of febuxostat excluding the cost of the drug.

### Return on investment

Taking a decision‐theoretic approach, the optimal sample size is that which maximizes the expectation of some objective function. Here, that objective function was taken to be the company ROI resulting from a particular trial outcome. This used a previously published and relatively simplistic model of ROI,^13^, ^35^ the additional inputs required are summarized in **Table** **2**. It was assumed that the price of febuxostat is set at the payer’s MRP, determined based on the posterior distribution of efficacy from a trial of size $\sum n_{j}$, as described in the previous section. It was further assumed that the company has a minimum price ($P_{\min}$), which, if above the payer’s MRP, results in termination of development and zero revenue. The cost of producing and marketing a year’s supply of febuxostat ($C_{\text{PM}}$) was included on a per‐patient basis. Total revenue was calculated for the *k*th trial design according to the MRP less the cost of production and marketing, then multiplied by the mean number of year’s supply of febuxostat per patient ($t_{j=2,k}$) and the number of patients who will receive febuxostat (*S*(*H*)) over some time horizon H.

(4) $${\text{ROI}}_{k}=\left\{\begin{array}{cc} \left[P_{2k}‐C_{\text{PM}}\right]t_{2k}\left(\psi_{k}\right)S\left(H\right)‐C_{\text{trial}}\left(\psi_{k}\right) & \text{if}\;P_{2k}\geq P_{\text{min}}\\ 0 & \text{if}\;P_{2k}<P_{\text{min}} \end{array}\right)$$

**Table 2:** Inputs values for payoff (return on investment) model

Cost group	Item (unit) (variable name)	Value
Drug development	Trial fixed cost (£) (*C*_TF_)	£5,000,000
	Trial variable cost (£) (C_TV_)	£20,000
	Production and marketing (£ per annum) (C_PM_)	£10
	Minimum price (£ per annum) (*P*_min_)	£70

Reimbursement authority	Cost effectiveness threshold (£) λ	£20,000
Marketing	Gout incidence (persons per annum) (*I*)^a^	100,000
	Market share (%) (*s*)	40
	Time horizon (years) (*H*)	10
	Deflation index (%) (*r*)	4

^a^ Incidence was halved in scenarios where the serum uric acid threshold for treatment was 8 mg/dL.

The methods used to calculate *S*(*H*) and the trial costs are presented in the **Supplementary Material**.

## RESULTS

Clinical trial simulation results are summarized in **Table** **3** in terms of the primary outcome of treatment response, defined as the percentage of subjects with a sUA trough concentration measurement of < 6 mg/dL on the last day of a trial. The mean response rate for allopurinol ranged from 11 to 59%. The minimum used a 300 mg dose, included only patients with baseline sUA > 8 mg/dL, and assumed less uncertainty on model inputs ($\psi_{5}$). The maximum used a 600 mg dose and included only patients with baseline sUA > 6 mg/dL. The mean response rate for febuxostat was less variable, ranging from 64 to 70%. The minimum used a dose of 80 mg, included only patients with baseline sUA > 8 mg/dL, and base case uncertainty on model inputs ($\psi_{2}$). The maximum used a dose of 120 mg and included only patients with baseline sUA > 6 mg/dL. The uncertainty in the response rate, shown as SD and 2.5th and 97.5th percentiles, was higher for allopurinol than febuxostat. The simulation results, therefore, indicate greater confidence in the prior estimate of febuxostat in a future trial than the comparator allopurinol.

**Table 3:** Clinical trial simulation results and prior distribution parameter values for the percentage of subjects with sUA < 6 mg/dL

Scenario	Design	Mean	SD	Percentiles		Beta parameters	
				2.5	97.5	Shape (α)	Scale (β)
Allopurinol arm							
Base case	ψ_1_	36	10.6	17	57	7.1	12.4
	ψ_2_	14	11.1	1	42	1.3	7.6
	ψ_3_	59	10.2	37	76	13.2	9
Reduced	ψ_4_	34	5.7	23	45	23.7	45.2
	ψ_5_	11	5	3	22	4.2	33.9
	ψ_6_	59	6.3	44	70	35.2	24.4
Febuxostat arm							
Base case	ψ_1_	67	6.3	51	76	36.4	17.6
	ψ_2_	64	9.6	38	75	15.3	8.6
	ψ_3_	70	4.8	58	77	62.8	26.9
Reduced	ψ_4_	68	5.2	55	75	53.4	25.1
	ψ_5_	65	7.3	46	74	27.1	14.4
	ψ_6_	70	4	61	76	89	37.5

sUA, serum uric acid.

**Figure 2:**
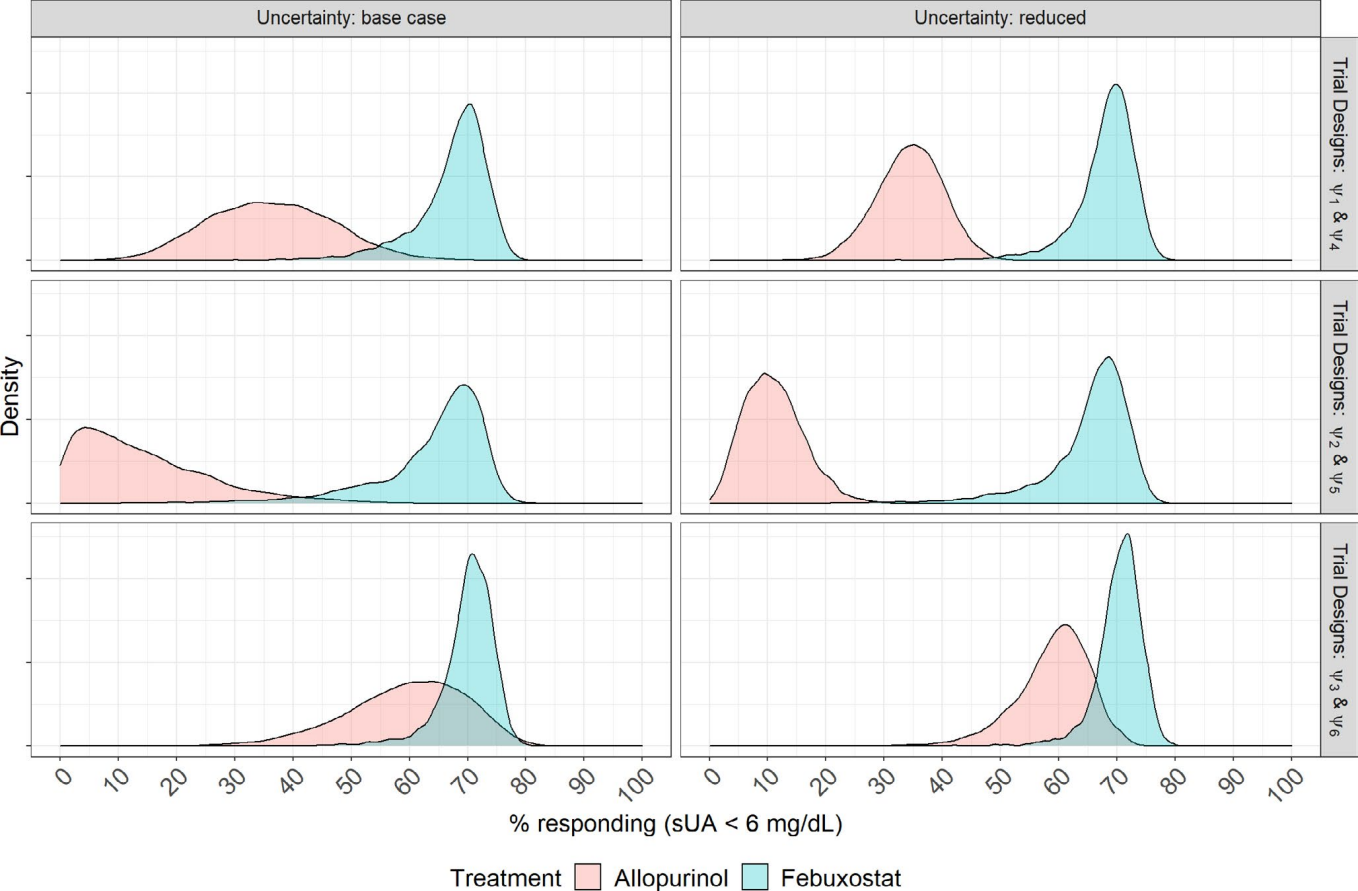
Simulated prior distributions of treatment effect (proportion of responders at sUA < 6 mg/dL) mg/dL. sUA, serum uric acid.

The distributions in response rate from the CTS are presented in **Figure** **2**, showing the relative effects of each treatment and the uncertainty for the different scenarios considered. We observe that the higher sUA threshold for inclusion onto the trial impacts allopurinol much more than the response rate for febuxostat. Using the higher doses of both drugs improves the response rate for allopurinol more than for febuxostat. Reducing the scale of uncertainty on input parameters has a noticeable impact on the width of distributions presented, however, considerable uncertainty in the simulated response rate remains.

**Figure 3:**
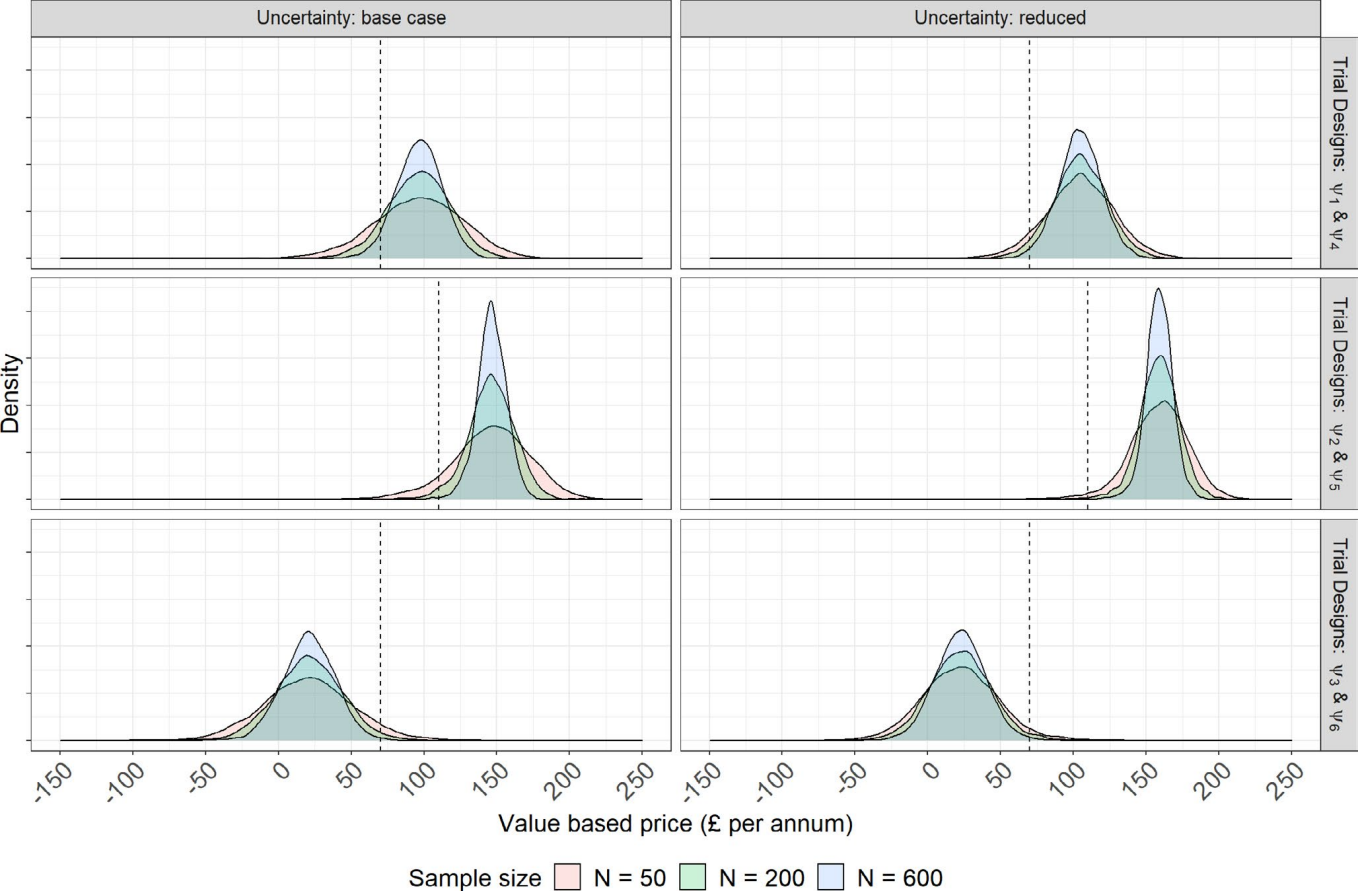
Distributions of maximum reimbursement prices for selected trial sample sizes.

**Figure** **3** shows the distribution of MRPs that were obtained at the end of stage 3 having performed Bayesian updating and calculated using costs and QALYs from the pharmacoeconomic model. Three of the eight sample sizes considered have been presented. As expected, the greater the separation between the distributions of response for allopurinol and febuxostat in **Figure** **2** the higher the predicted MRPs. This reflects the assumed willingness of payers to pay higher prices for greater benefit relative to the standard of care, allopurinol, in line with its cost‐effectiveness threshold. We observed reduced uncertainty in the predicted MRPs for larger sample sizes, and in simulations with reduced uncertainty in CTS inputs.

In the final stage, the predicted MRPs were used as inputs to a model of company ROI, which are presented in **Table** **4** for each design and sample size. The designs $\psi_{1}$ and $\psi_{4}$ yield the greatest expected ROI overall, due to the assumed larger patient population for a lower sUA threshold for treatment of 6 mg/dL. Design $\psi_{4}$ is higher than $\psi_{1}$ and is maximized at a lower sample size, 150 per arm compared with 300 per arm. The expected ROI for $\psi_{2}$ was a maximum for 200 subjects per arm and for $\psi_{5}$ only 50 subjects per arm, however, such a small trial is unlikely to be sufficient in practice when considering safety outcomes. For the higher dosage designs, $\psi_{3}$ and $\psi_{6}$, the expected ROI is negative for sample sizes ≥ 150 and 100, respectively.

**Table 4:** Mean ROI for six trial design scenarios and eight sample sizes

Scenario	Design	ROI (£) by sample size							
		50	100	150	200	300	400	500	600
Base case	ψ_1_	352,033,215	356,945,139	359,499,211	361,877,215	**362,712,498**	362,369,826	361,350,036	359,065,887
	ψ_2_	288,802,211	293,836,616	295,727,330	**296,208,230**	294,849,790	291,852,035	288,269,114	284,551,435
	ψ_3_	**12,206,729**	2,306,807	−3,223,324	−7,494,414	−13,870,284	−18,990,268	−23,669,712	−27,889,227
Reduced	ψ_4_	410,975,796	412,957,108	**413,323,108**	412,895,094	411,246,682	408,795,157	405,639,530	402,603,965
	ψ_5_	**331,655,061**	331,264,110	330,170,059	328,671,589	325,115,645	321,392,535	317,531,941	313,525,726
	ψ_6_	**5,322,523**	−270,309	−4,165,476	−7,347,405	−13,518,429	−18,527,435	−23,214,286	−27,863,108

The sample size with the highest ROI for each trial design has been highlighted.

ROI, return on investment.

## DISCUSSION

This study has demonstrated an approach to trial design and sample size calculation that is based on value of information analysis, or fully Bayesian trial design, in which the optimal sample size is that which results in the greatest expected ROI to the pharmaceutical company. However, contrasting with previous analyses, we used pharmacometric models to simulate prior distributions of treatment effect for the investigational drug and comparator under varying trial designs defined in terms of doses, patient characteristics, and uncertainty on input parameters. The prior distributions can, therefore, be developed in a transparent way that represents the specific conditions under which a drug will be used in a proposed trial. This would be advantageous in situations where changes in treatment dose, regimen, patients, or comparators, means that evidence from earlier studies is less likely to provide a reliable estimate of treatment effect in future studies. It also allows other aspects of trial design to be examined at the same time as performing sample size calculations.

We illustrated this interdisciplinary approach using a case study of designing a single phase III trial of febuxostat versus allopurinol. The greatest expected ROI was predicted to occur with reduced uncertainty in PK/PD inputs, suggesting that it may be of value to gather further evidence relating to the pharmacology of the drugs before proceeding to large scale phase III testing. The optimal sample size for this scenario was also lower than the corresponding design with a higher level of input uncertainty. The results also showed that if higher doses of the comparator were necessary, then expected ROI would only be positive if very small trials were practicable.

Clinical trial simulation has typically been implemented within a model‐informed drug development context,^36^ for example, to support design decisions based on predicted performance in statistical tests, in order to meet efficacy and safety objectives and obtain regulatory approval.^37^ Linking CTS results to an economic model designed to represent the reimbursement authorities approach to drug pricing is a natural extension that is consistent with an model‐informed drug development approach. Poland and Wada^38^ presented a combined PK/PD and economic model to compare alternative dose regimens, including models for non‐adherence. However, although the drug price was linked to the drug’s simulated efficacy and safety, it did not consider whether the drug would be reimbursed at these prices. There are further examples of linking pharmacometric and pharmacoeconomic models^39^, ^40^ but these do not explicitly consider trial design nor do they consider the relationship between potential pricing and trial results.

For the purpose of demonstrating the value of this interdisciplinary approach, the decision problem was simplified. In reality, there may be the need to consider the design and value of multiple phase III trials, as was the case for febuxostat.^41^ There are also multiple markets to consider and, therefore, multiple payers and reimbursement authorities with differing approaches to valuing medicines, with the additional complication that prices cannot be set in each market independently.^42^ Others have adopted more realistic decision contexts by, for example, linking the market share to trial outcomes,^12^, ^38^ considering multistage/adaptive trials,^10^, ^43^ and assuming imperfect implementation of a policy decision.^44^ As has been observed in previous research in this area,^13^ the value of information approach does not easily apply to a free market setting unless there is a means of linking pricing and sales volumes to trial outcomes. This general basic framework could be adapted to real‐world decision problems, building in additional complexity as a particular case study requires.

This study assumed that regulatory approval would be granted, because both treatments were assumed safe and effective. It would be possible to define a utility function that incorporates both the probabilities of regulatory approval and reimbursement as a function of trial results.^12^ However, this implies that a regulator would accept an analysis of trials using informative priors such as in this study. It may be possible to avoid this by incorporating frequentist hypothesis test constrains within the Bayesian utility function.^45^

Furthermore, this study has assumed that the phase III trial is only used to inform the estimate of treatment efficacy, implying that the treatments considered are equivalent in terms of safety. This method could be extended to include simulation of safety outcomes, in order to predict benefit‐risk assessments in cases where treatments may differ in terms of safety.^46^ This also applies to other data that may be collected from a proposed trial, such as evidence regarding the utility of different health states or resource use that is sometimes derived from pivotal studies. The approach we have described also relies on being able to make valid predictions of treatment effects in a phase III trial population using models developed from earlier studies.

The technical challenge of performing a linked CTS and economic modeling exercise may be considered a limitation of this approach, however, much of the modeling effort already takes place within industry; PK/PD models are used extensively during drug development and economic modeling is often required in order to secure reimbursement. The combined models incorporate a large number of input parameters and also require many simplifying assumptions, to which the results may be sensitive. This may be considered both a strength and a limitation, because although it reduces our confidence in the model accuracy, it does provide a framework to understand the impacts of alternative assumptions and parameter uncertainty.

Individual level CTS and economic modeling is likely to require significant computing resources to implement within a reasonable timeframe. This study benefited from access to a supercomputing cluster, although other applications may not be as computationally intensive. As this case study was used primarily to illustrate the methods, some parameters and parameter uncertainty were assumed rather than estimated, and the important issue of correlation between input parameters was not considered. Finally, the model of dose implementation that was used in which daily doses are missed at random independent of whether previous doses were taken is overly simplistic. Alternatively, more sophisticated models may be used or replaced with real‐world adherence data.

We have presented an interdisciplinary framework for trial design and sample size calculations linking several modeling approaches that are all implemented separately at different stages during drug development. It enables uncertainty to be propagated from aspects of drug pharmacology through to the predicted return on investment, where drug prices are contingent on the results of phase III testing. Such an approach may have value in supporting trial design decisions and in identifying costly sources of uncertainty, and thus inform future research and development strategies.

## Funding

This study was funded by the MRC Network of Hubs for Trial Methodological Research (HTMR), reference number MR/L004933/1‐Q25, and the MRC North‐West HTMR, reference number MR/K025635/1.

## Conflict of Interest

The authors declared no competing interests for this work.

## Author Contributions

D.H.‐M. and D.A.H. wrote the manuscript. D.H.‐M. and D.A.H. designed the research. D.H.‐M. performed the research. D.H.‐M. analyzed the data.

## Supporting information

Supplementary Material
