# Supplementary material for: Combining Model‐Based Clinical Trial Simulation, Pharmacoeconomics, and Value of Information to Optimize Trial Design
Source: CPT Pharmacometrics Syst Pharmacol. 2020 Dec 31;10(1):75–83. doi: 10.1002/psp4.12579 (PMC7825194; doi:10.1002/psp4.12579)
Supplement: Supplementary file 1 — Supplementary Material [file PSP4-10-75-s001.pdf]

Combining model-based clinical trial simulation, pharmacoeconomics and value of information to optimise trial design: SUPPLEMENTARY MATERIAL

Contents

1 Appendix 1: Pharmacometric Model Overview **2**

1.1 Pharmacokinetic Models 2

1.2 Pharmacodynamic Model Structure 2

1.3 Pharmacodynamic Model Mathematical Description 3

2 Appendix 2: Clinical Trial Simulation Base Case Uncertainty Scenario Inputs **5**

2.1 Trial Conduct Inputs 5

2.2 Pharmacokinetic Inputs 6

2.3 Pharmacodynamic Inputs 8

3 Appendix 3: Clinical Trial Simulation Reduced Uncertainty Scenario Inputs **9**

3.1 Trial Conduct Inputs 9

3.2 Pharmacokinetic Inputs 10

3.3 Pharmacodynamic Inputs 11

4 Appendix 4: Pharmacoeconomic Model and Inputs **12**

4.1 Pharmacoeconomic Markov Model Structure 12

4.2 Pharmacoeconomic Inputs 13

5 Appendix 5: Return on Investment Model **14**

1 Appendix 1: Pharmacometric Model Overview

1.1 Pharmacokinetic Models

Allopurinol used a one-compartment pharmacokinetic (PK) model with first-order absorption and elimination. There are covariate sub-models for clearance and volume of distribution and are inter-individual variability models for clearance and absorption. Parameter values and covariate model equations are given in Sections 2.2 and 3.2.

Febuxostat used a two-compartment PK model with first-order absorption and elimination. There are covariate sub-models for clearance and inter-individual variability models for clearance and absorption. Parameter values and covariate model equations are given in Sections 2.2 and 3.2.

1.2 Pharmacodynamic Model Structure

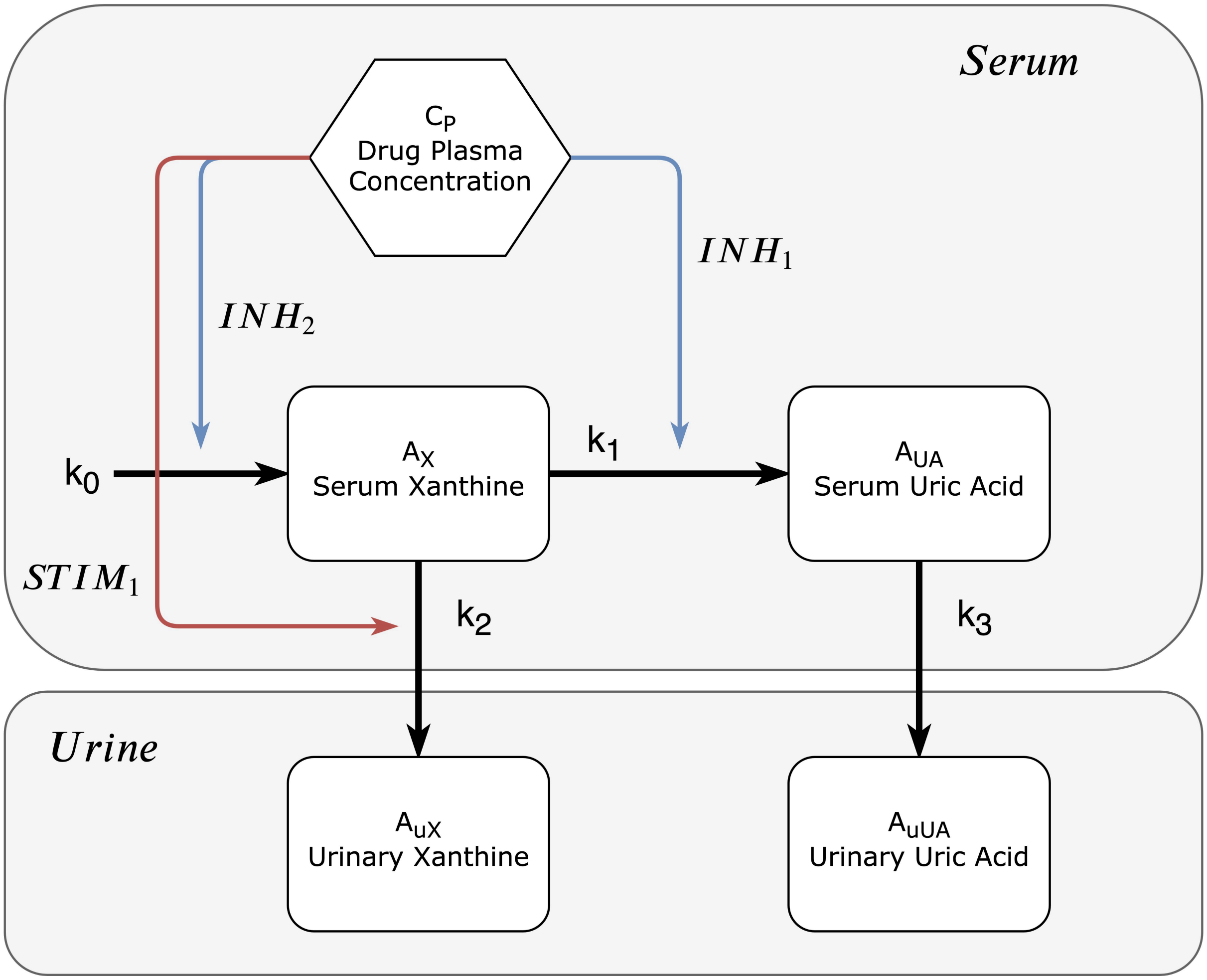

Figure 1: Structure of the semi-mechanistic pharmacodynamic model

1.3 Pharmacodynamic Model Mathematical Description

The indirect response drug pharmacodynamic equations are shown below. Parameter input values are given in appendix 2 and 3.

Allopurinol pharmacodynamic equations

$INH_1 = 1 - \frac{IMAX_A * C_A(t)^{\lambda}}{IC50_{1_A}^{\lambda} + C_A(t)^{\lambda}}$
$INH_2 = 1 - \frac{IMAX_A * C_A(t)^{\lambda}}{IC50_{2_A}^{\lambda} + C_A(t)^{\lambda}}$

Febuxostat pharmacodynamic equations

$INH_1 = 1 - \frac{IMAX_F * C_F(t)}{IC50_{1_F} + C_F(t)}$
$INH_2 = 1 - \frac{IMAX_F * C_F(t)}{IC50_{2_F} + C_F(t)}$
$STIM_1 = 1 + \frac{EMAX * C_F(t)}{EC50 + C_F(t)}$

INH_1 and INH_2 are inhibitory pharmacodynamic model drug functions; $STIM_1$ is a stimulatory pharmacodynamic model drug function; $C_F(t)$ and $C_A(t)$ are the plasma concentrations of febuxostat and allopurinol, respectively; $IMAX$ is the maximum possible level of inhibition in the pharmacodynamic drug models INH_1 and INH_2; $IC50_1$ and $IC50_2$ are the drug concentrations corresponding to 50% of the maximum possible level of inhibition in the pharmacodynamic drug models INH_1 and INH_2 respectively; $EC50$ is the drug concentration corresponding to 50% of the maximum possible level of stimulation in the pharmacodynamic drug model $STIM_1$; $EMAX$ is the maximum possible level of stimulation in the pharmacodynamic drug model $STIM1$; λ is the hill coefficient.

System Model

The rate of change in each of the four pharmacodynamic model compartments:

$\frac{dA_X}{dt} = k_0 * INH_2 - k_1 * INH_1 * A_X - K_2 * STIM_1 * A_X$
$\frac{dA_{UA}}{dt} = k_1 * INH_1 * A_X * r - k_3 * A_{UA}$
$\frac{dA_{uX}}{dt} = k_2 * STIM_1 * A_X$
$\frac{dA_{uUA}}{dt} = k_3 * A_{UA}$

A_X and A_{UA} are the total time varying amounts of xanthine and uric acid in serum respectively; A_{uX} and A_{uUA} are the total time-varying amounts of xanthine and uric acid in urine respectively; k_0, k_1, k_2 and k_3 are the rate parameters for the production of xanthine, xanthine to uric acid conversion, removal of xanthine to urine and removal of uric acid to urine, respectively. r is the ratio of uric acid to xanthine molar masses.

The pharmacodynamic model rate contsants can be calculated at steady-state according to:

$k_0 = k_1 * B_X + k_2 * B_X$

$k_1 = \frac{k_3 * B_{UA}}{B_X * (M_{UA}/M_X)}$

$k_2 = \frac{CL_X}{V_X}$

$k_3 = \frac{CL_{UA}}{V_{UA}}$

Where B_{UA} is the baseline amount of uric acid; B_X is the baseline amount of xanthine; CL_{UA} is the renal clearance of uric acid; CL_X is the renal clearance of xanthine; V_{UA} is the volume of uric acid distribution; V_X is the volume of xanthine distribution; M_{UA} and M_X are the molar masses of uric acid and xanthine respectively.

2 Appendix 2: Clinical Trial Simulation Base Case Uncertainty Scenario Inputs

2.1 Trial Conduct Inputs

Table 1: Trial Conduct Parameter Inputs

Parameter	Mean	Units	CV%/100	Distribution
**Adherence model: allopurinol**				
$\lambda_{weibull_A}(dropout)$	0.0032	na	0.1	beta
$k_{weibull_A}(dropout)$	0.8	na	0	na
μ_{IMP_A}	0.9	na	0.1	beta
σ_{IMP_A}	0.1	na	0	na
**Adherence model: febuxostat**				
$\lambda_{weibull_F}(dropout)$	0.005	na	0.1	beta
$k_{weibull_F}(dropout)$	0.8	na	0	na
μ_{IMP_F}	0.9	na	0.1	beta
σ_{IMP_F}	0.1	na	0	na
**Cohort recruitment inputs**				
μ_{SUA}	8.34	mg/dL	0	na
μ_{WT}	100	kg	0	na
μ_{AGE}	60	years	0	na
μ_{VUA}	154	dL	0	na
ω_{SUA}	0.258	na	0	na
ω_{WT}	0.1	na	0	na
ω_{AGE}	0.252	na	0	na
ω_{VUA}	0.1	na	0	na

Subject specific dose implementation calculated according to:

$IMP_i = Beta(\alpha, \beta)$

$\alpha = (\frac{1-\mu_{IMP}}{\sigma^2_{IMP}} - \frac{1}{\mu_{IMP}})\mu^2_{IMP}$

$\beta = \alpha(\frac{1}{\mu_{IMP}} - 1)$

Subject specific attribute calculated according to:

$\mu_i = \mu * e^{\eta}$

$\eta \sim N(0, \omega)$

2.2 Pharmacokinetic Inputs

Population typical values for parameters are first sampled using the column CV% according to (e.g. CL_F):

$\sigma = CV * CL_F$

$A = log(CL_F/(\sqrt{1 + (\sigma/CL_F)^2}))$

$B = \sqrt{log(1 + (\sigma/CL_F)^2)}$

$CL_{F_j} \sim LogNormal(A, B)$ for the jth CTS replicate.

Table 2: Pharmacokinetic parameters for allopurinol (1-compartment model)

Parameter	Mean	Units	CV%/100	Distribution
CL_A	13.2	dL/h	0.2	logN
VC_A	416	dL	0.2	logN
KA_A	1.09	h^{-1}	0.2	logN
$CRCL_{ref}$	100	dL/h	0	na
WT_{ref}	70	kg	0	na
τ_{CRCL}	0.587	na	0.2	logN
τ_{WT}	0.74	na	0.2	logN
ω_{CL_A}	0.252	dL/h	0.2	logN
ω_{KA_A}	0.589	h^{-1}	0.2	logN

Parameter values for specific subject i obtained according to:

$CL_{A_i} = CL_A * (CRCL_i/CRCL_{ref})^{\tau_{CRCL}} * (WT_i/WT_{ref})^{\tau_{WT}} * e^{\eta_{CL_i}}$

$VC_{A_i} = VC_A * (WT_i/WT_{ref})$

$KA_{A_i} = KA_A * e^{\eta_{KA_i}}$

Where: $\eta_{X_i} \sim N(0, \omega_X)$ for a fixed-effect parameter X.

Table 3: Pharmacokinetic prameters for febuxostat (2-compartment model)

Parameter	Mean	Units	CV%/100	Distribution[1]
CL_F	49.3	dL/h	0.2	logN
KA_F	13.7	h^{-1}	0.2	logN
VC_F	322	dL	0.2	logN
VP_F	222	dL	0.2	logN
Q_F	55.7	dL/h	0.2	logN
θ_{CRCL}	0.142	na	0.2	logN
θ_{WT}	0.155	na	0.2	logN
ω_{CL_F}	0.033	dL/h	0.2	logN
ω_{KA_F}	1.41	h^{-1}	0.2	logN

Parameter values for specific subject i obtained according to:

$CL_{F_i} = CL_F + CRCL_i * \theta_{CRCL} + WT_i * \theta_{WT}) * \theta_i * e^{\eta_{CL_{F_i}}}$

$KA_{F_i} = KA_F * e^{\eta_{KA_{F_i}}}$

Where: $\eta_{X_i} \sim N(0, \omega_X)$ for a fixed-effect parameter X.

2.3 Pharmacodynamic Inputs

Table 4: Pharmacodynamic prameters for allopurinol

Parameter	Mean	Units	CV%/100	Distribution
$IMAX_A$	0.7231	na	0	na
$IC50_{1_A}$	0.0129	mg/dL	0.2	logN
$IC50_{2_A}$	1.5068	mg/dL	0.2	logN
λ	1.3652	na	0.2	logN
$\omega_{IC50_{1_A}}$	0.2	na	0.2	logN
$\omega_{IC50_{2_A}}$	0.2	na	0.2	logN

Table 5: Pharmacodynamic prameters for febuxostat

Parameter	Mean	Units	CV%/100	Distribution
EMAX	3	na	0	na
$EC50$	0.001	mg/dL	0.2	logN
$IMAX_F$	1	na	0	na
$IC50_{1_F}$	0.0011	mg/dL	0.2	logN
$IC50_{2_F}$	0.132	mg/dL	0.2	logN
$\omega_{IC50_{1_F}}$	0.2	na	0.2	logN
$\omega_{IC50_{2_F}}$	0.2	na	0.2	logN

3 Appendix 3: Clinical Trial Simulation Reduced Uncertainty Scenario Inputs

3.1 Trial Conduct Inputs

Table 6: Trial Conduct Parameter Inputs

Parameter	Mean	Units	CV%/100	Distribution
**Adherence model: allopurinol**				
$\lambda_{weibull_A}(dropout)$	0.0032	na	0.1	beta
$k_{weibull_A}(dropout)$	0.8	na	0	na
μ_{IMP_A}	0.9	na	0.1	beta
σ_{IMP_A}	0.1	na	0	na
**Adherence model: febuxostat**				
$\lambda_{weibull_F}(dropout)$	0.005	na	0.1	beta
$k_{weibull_F}(dropout)$	0.8	na	0	na
μ_{IMP_F}	0.9	na	0.1	beta
σ_{IMP_F}	0.1	na	0	na
**Cohort recruitment inputs**				
μ_{SUA}	8.34	mg/dL	0	na
μ_{WT}	100	kg	0	na
μ_{AGE}	60	years	0	na
μ_{VUA}	154	dL	0	na
ω_{SUA}	0.258	na	0	na
ω_{WT}	0.1	na	0	na
ω_{AGE}	0.252	na	0	na
ω_{VUA}	0.1	na	0	na

Subject specific dose implementation calculated according to:

$IMP_i = Beta(\alpha, \beta)$

$\alpha = (\frac{1-\mu_{IMP}}{\sigma^2_{IMP}} - \frac{1}{\mu_{IMP}})\mu^2_{IMP}$

$\beta = \alpha(\frac{1}{\mu_{IMP}} - 1)$

Subject specific attribute calculated according to:

$\mu_i = \mu * e^{\eta}$

$\eta \sim N(0, \omega)$

3.2 Pharmacokinetic Inputs

Population typical values for parameters are first sampled using the column CV% according to (e.g. CL_F):

$\sigma = CV * CL_F$

$A = log(CL_F/(\sqrt{1 + (\sigma/CL_F)^2}))$

$B = \sqrt{log(1 + (\sigma/CL_F)^2)}$

$CL_{F_j} \sim LogNormal(A, B)$ for the jth CTS replicate.

Table 7: Pharmacokinetic parameters for allopurinol (1-compartment model)

Parameter	Mean	Units	CV%/100	Distribution
CL_A	13.2	dL/h	0.05	logN
VC_A	416	dL	0.05	logN
KA_A	1.09	h^{-1}	0.05	logN
$CRCL_{ref}$	100	dL/h	0	na
WT_{ref}	70	kg	0	na
τ_{CRCL}	0.587	na	0.05	logN
τ_{WT}	0.74	na	0.05	logN
ω_{CL_A}	0.252	dL/h	0.05	logN
ω_{KA_A}	0.589	h^{-1}	0.05	logN

$CL_{A_i} = CL_A * (CRCL_i/CRCL_{ref})^{\tau_{CRCL}} * (WT_i/WT_{ref})^{\tau_{WT}} * e^{\eta_{CL_i}}$

$VC_{A_i} = VC_A * (WT_i/WT_{ref})$

$KA_{A_i} = KA_A * e^{\eta_{KA_i}}$

Where: $\eta_{X_i} \sim N(0, \omega_X)$ for a fixed-effect parameter X.

Table 8: Pharmacokinetic prameters for febuxostat (2-compartment model)

Parameter	Mean	Units	CV%/100	Distribution[1]
CL_F	49.3	dL/h	0.05	logN
KA_F	13.7	h^{-1}	0.05	logN
VC_F	322	dL	0.05	logN
VP_F	222	dL	0.05	logN
Q_F	55.7	dL/h	0.05	logN
θ_{CRCL}	0.142	na	0.05	logN
θ_{WT}	0.155	na	0.05	logN
ω_{CL_F}	0.033	dL/h	0.05	logN
ω_{KA_F}	1.41	h^{-1}	0.05	logN

Parameter values for specific subject i obtained according to:

$CL_{F_i} = CL_F + CRCL_i * \theta_{CRCL} + WT_i * \theta_{WT}) * \theta_i * e^{\eta_{CL_{F_i}}}$

$KA_{F_i} = KA_F * e^{\eta_{KA_{F_i}}}$

Where: $\eta_{X_i} \sim N(0, \omega_X)$ for a fixed-effect parameter X.

3.3 Pharmacodynamic Inputs

Table 9: Pharmacodynamic prameters for allopurinol

Parameter	Mean	Units	CV%	Distribution
$IMAX_A$	0.7231	na	0	na
$IC50_{1_A}$	0.0129	mg/dL	0.1	logN
$IC50_{2_A}$	1.5068	mg/dL	0.1	logN
λ	1.3652	na	0.1	logN
$\omega_{IC50_{1_A}}$	0.2	na	0.1	logN
$\omega_{IC50_{2_A}}$	0.2	na	0.1	logN

Table 10: Pharmacodynamic prameters for febuxostat

Parameter	Mean	Units	CV%	Distribution
EMAX	3	na	0	na
$EC50$	0.001	mg/dL	0.1	logN
$IMAX_F$	1	na	0	na
$IC50_{1_F}$	0.0011	mg/dL	0.1	logN
$IC50_{2_F}$	0.132	mg/dL	0.1	logN
$\omega_{IC50_{1_F}}$	0.2	na	0.1	logN
$\omega_{IC50_{2_F}}$	0.2	na	0.1	logN

4 Appendix 4: Pharmacoeconomic Model and Inputs

4.1 Pharmacoeconomic Markov Model Structure

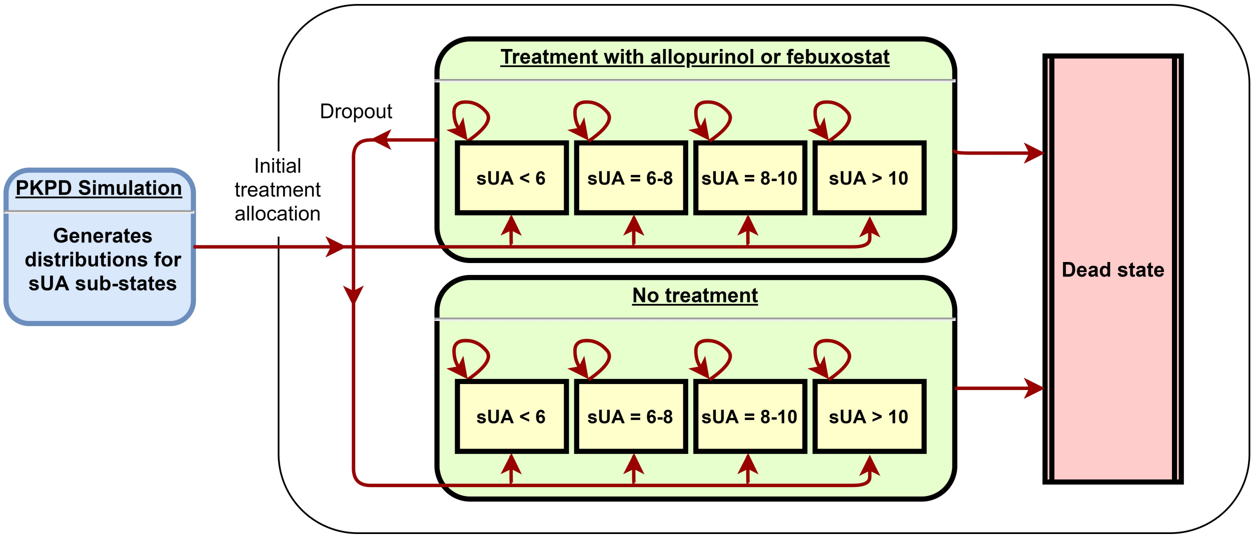

Figure 2: Structure of economic simulation model

4.2 Pharmacoeconomic Inputs

Table 11: Cost inputs

Parameter	Mean	Units
Allopurinol 300 mg q.d.	2.44	£ per cycle
Colchicine 0.5 mg q.d.	28.56	£ per cycle
Treatment monitoring visit	153.07	£ per cycle
Flare treatment cost	11.38	£ per cycle

Table 12: Serum uric acid concentration to flare frequency mapping

	sUA concentration category (mg/dL)			
Annual flare number	< 6	6 - 8	8 - 10	> 10
0	0.371	0.08	0.171	0.171
1-2	0.452	0.40	0.257	0.257
3	0.065	0.24	0.114	0.114
4-5	0.081	0.20	0.257	0.257
6+	0.032	0.08	0.200	0.200

Table 13: Health state utilities by frequency of gout flares

	Annual number of flares				
Disease state	0	1-2	3	4-5	6+
Tophi present	0.725	0.719	0.701	0.701	0.663
Tophi not present	0.778	0.759	0.735	0.735	0.710

Table 14: Additional inputs

Parameter	Value
Treatment induced flare (slope)	0.0119
Treatment induced flare (constant)	0.4408
Proportion with tophi (%)	18

5 Appendix 5: Return on Investment Model

As described in the manuscript, the company return on investment is given by:

$$ROI_k = \begin{cases} [P_{2k} - C_{PM}]t_{2k}(\psi_k)S(H) - C_{trial}(\psi_k), & \text{if } P_{2k} \geqslant P_{min}. \\ 0, & \text{if } P_{2k} < P_{min}. \end{cases}$$

The sub-models for S(H) and trial costs are presentenced in the supplementary material. Drawing on the work of Hoyle[1] and Willan[2], we predicted the number of patients who will receive febuxostat as a function of the annual disease incidence I, the market share s and a depreciation factor used to model real pharmaceutical price deflation v according to:

$S_F(H) = \sum_{h=1}^{H} Isv^h$ where $v^h = 1/(1+r)^h$ and r is the deflation index. The time horizon (H) was taken to be the number of years of patent protection (or market exclusivity) remaining when the drug reaches the market, which we have assumed to be 10 years. The values assumed for these inputs are given in Table 2. Finally, the cost of the trial was decomposed into fixed and variable elements, with the latter being proportional to the number of patients recruited. The separation of trial costs is shown below, where n_1 and n_2 are the numbers of patients recruited to the allopurinol and febuxostat trial arms respectively.

$C_{trial} = C_{TF} + (n_1 + n_2) * C_{TV}$

1. Hoyle, M. Accounting for the drug life cycle and future drug prices in cost-effectiveness analysis. Pharmacoeconomics 29, 1–15 (2011).
2. Willan, A. R. Optimal sample size determinations from an industry perspective based on the expected value of information. Clin. Trials 5, 587–594 (2008).
